# ^44^Sc for labeling of DOTA- and NODAGA-functionalized peptides: preclinical in vitro and in vivo investigations

**DOI:** 10.1186/s41181-016-0013-5

**Published:** 2016-05-05

**Authors:** Katharina A. Domnanich, Cristina Müller, Renata Farkas, Raffaella M. Schmid, Bernard Ponsard, Roger Schibli, Andreas Türler, Nicholas P. van der Meulen

**Affiliations:** 1 Laboratory of Radiochemistry, Paul Scherrer Institute, CH-5232 Villigen-PSI, Switzerland; 2 Department of Chemistry and Biochemistry, University of Bern, 3012 Bern, Switzerland; 3 Center for Radiopharmaceutical Sciences ETH-PSI-USZ, Paul Scherrer Institute, 5232 Villigen-PSI, Switzerland; 4 Department of Chemistry and Applied Biosciences, ETH Zurich, 8093 Zurich, Switzerland; 5 SCK.CEN, BR2 Reactor, 2400 Mol, Belgium

**Keywords:** ^44^Sc, PET, Imaging, Stability, DOTA-RGD, NODAGA-RGD, DOTA-NOC, NODAGA-NOC, ^68^Ga, AR42J, U87MG

## Abstract

**Background:**

Recently, ^44^Sc (T_1/2_ = 3.97 h, Eβ^+^_av_ = 632 keV, I = 94.3 %) has emerged as an attractive radiometal candidate for PET imaging using DOTA-functionalized biomolecules. The aim of this study was to investigate the potential of using NODAGA for the coordination of ^44^Sc. Two pairs of DOTA/NODAGA-derivatized peptides were investigated in vitro and in vivo and the results obtained with ^44^Sc compared with its ^68^Ga-labeled counterparts.

DOTA-RGD and NODAGA-RGD, as well as DOTA-NOC and NODAGA-NOC, were labeled with ^44^Sc and ^68^Ga, respectively. The radiopeptides were investigated with regard to their stability in buffer solution and under metal challenge conditions using Fe^3+^ and Cu^2+^. Time-dependent biodistribution studies and PET/CT imaging were performed in U87MG and AR42J tumor-bearing mice.

**Results:**

Both RGD- and NOC-based peptides with a DOTA chelator were readily labeled with ^44^Sc and ^68^Ga, respectively, and remained stable over at least 4 half-lives of the corresponding radionuclide. In contrast, the labeling of NODAGA-functionalized peptides with ^44^Sc was more challenging and the resulting radiopeptides were clearly less stable than the DOTA-derivatized matches. ^44^Sc-NODAGA peptides were clearly more susceptible to metal challenge than ^44^Sc-DOTA peptides under the same conditions. Instability of ^68^Ga-labeled peptides was only observed if they were coordinated with a DOTA in the presence of excess Cu^2+^. Biodistribution data of the ^44^Sc-labeled peptides were largely comparable with the data obtained with the ^68^Ga-labeled counterparts. It was only in the liver tissue that the uptake of ^68^Ga-labeled DOTA compounds was markedly higher than for the ^44^Sc-labeled version and this was also visible on PET/CT images. The ^44^Sc-labeled NODAGA-peptides showed a similar tissue distribution to those of the DOTA peptides without any obvious signs of in vivo instability.

**Conclusions:**

Although DOTA revealed to be the preferred chelator for stable coordination of ^44^Sc, the data presented in this work indicate the possibility of using NODAGA in combination with ^44^Sc. In view of a clinical study, thorough investigations will be necessary regarding the labeling conditions and storage solutions in order to guarantee sufficient stability of ^44^Sc-labeled NODAGA compounds.

**Electronic supplementary material:**

The online version of this article (doi:10.1186/s41181-016-0013-5) contains supplementary material, which is available to authorized users.

## Background

^44^Sc is a novel radiometal which is attractive for positron emission tomography (PET) imaging, due to the emission of positrons with a high branching ratio (Eβ^+^_av_ = 632 keV, I = 94.3 %) (Rösch 2012; Müller et al. 2013). Its physical half-life of 3.97 h enables the acquisition of PET images several hours after injection of the ^44^Sc-radiopharmaceutical and is of particular interest for application with biomolecules, providing slower kinetic profiles (Chakravarty et al. 2014). Importantly, application of ^44^Sc would allow a centralized production of radiopharmaceuticals, followed by transportation to more remote hospitals (van der Meulen et al. 2015).

^44^Sc is thought to be useful as a diagnostic match to therapeutic radionuclides with similar chemical properties, such as ^90^Y and ^177^Lu (Müller et al. 2013). Most interesting, however, would be to use ^44^Sc in combination with its therapeutic counterpart ^47^Sc, which provides excellent β^−^-decay properties for radionuclide therapy (Eβ^−^_av_ = 162 keV, T_1/2_ = 3.35 d). The potential of ^44^Sc/^47^Sc as a theragnostic pair has been demonstrated recently in a preclinical pilot study with tumor-bearing mice (Müller et al. 2014).

A crucial requirement for the application of radiopharmaceuticals is the formation of a thermodynamically stable and kinetically inert complex of the radiometal with a suitable chelator, which is then linked to the targeting agent (Majkowska-Pilip & Bilewicz 2011). The coordination of Sc(III) and Ga(III) has previously been investigated with several macrocyclic polyaminocarboxylic chelators, including 1,4,7,10-tetraazacyclododecane-1,4,7,10-tetraacetic acid (DOTA) and 1,4,7-triazacyclononane-1,4,7-triacetic acid (NOTA) (Majkowska-Pilip & Bilewicz 2011; Huclier-Markai et al. 2011; Notni et al. 2012). The studies were performed with ^nat^Sc(III) stock solutions containing ^46^Sc as tracer and the sole chelator without an attached biomolecule. As a result of these investigations, it was found that Sc(III) forms complexes with both chelators, DOTA and NOTA, however, the stability of Sc-DOTA was superior to Sc-NOTA complexes (Majkowska-Pilip & Bilewicz 2011; Huclier-Markai et al. 2011). On the other hand, Ga(III) displays a reverse behavior, forming more stable complexes with NOTA than with DOTA (Majkowska-Pilip & Bilewicz 2011; Notni et al. 2012). The DOTA-chelator provides eight coordination sites, which are all coordinated by Sc(III), whereas NOTA can only form six coordinative bonds. Due to the higher denticity the Sc-DOTA complex is believed to be thermodynamically more stable than the Sc-NOTA complex (Huclier-Markai et al. 2011; Port et al. 2008). Due to the preference of Ga(III) for the coordination number six, all coordination sites of NOTA are used, while two sites of DOTA remain uncoordinated in a Ga-DOTA-complex (Majkowska-Pilip & Bilewicz 2011; Viola-Villegas & Doyle 2009).

Despite the reduced stability of a Ga-DOTA complex compared to a Ga-NOTA and Ga-1,4,7-triazacyclononane,1-glutaric acid-4,7-acetic acid (NODAGA) complex, there are several examples of ^68^Ga-DOTA labeled peptides which showed promising in vivo properties. The ^68^Ga-labeled somatostatin receptor analogues ^68^Ga-DOTA-TOC, ^68^Ga-DOTA-TATE and ^68^Ga-DOTA-NOC represent the most prominently applied radiopharmaceuticals in clinical studies for imaging of neuroendocrine tumors (Kwekkeboom et al. 2010). Recent PET imaging studies in patients using the α_v_β_3_ integrin-targeting radiotracer ^68^Ga-NOTA-RGD indicated promising results (Kim et al. 2012; Choi et al. 2013; Yoon et al. 2014). These reports demonstrate that ^68^Ga is successfully used in clinics with both DOTA- and NOTA/NODAGA-derivatized targeting agents.

The stable complexation of ^44^Sc with DOTA initiated a number of preclinical studies with a range of DOTA-derivatized biomolecules, including bombesin analogues (Koumarianou et al. 2012), puromycin (Eigner et al. 2013), folate-conjugates (Müller et al. 2013) and dimeric cyclic RGD peptides (Hernandez et al. 2014). To date, all research with regard to the in vivo and in vitro behavior of ^44^Sc-labeled radiopharmaceuticals was performed with DOTA chelators connected to the respective targeting agent. The only exception to our knowledge was a study in which an EGFR-targeted antibody was labeled with ^44^Sc using a CHX-A-DTPA (N-[(R)-2-amino-3-(para-isothiocyanato-phenyl)propyl]trans-(S,S)-cyclohexane-1,2-diamine *N,N,N’,N”N”*-pentaacetic acid) chelator (Chakravarty et al. 2014). The question on whether or not a NOTA or NODAGA chelator would be suited for ^44^Sc-labeling has remained unclear thus far. Using these chelators for coordination of ^44^Sc would be of interest in combination with radiopharmaceuticals where only the NOTA- or NODAGA-derivatized species are available for clinical studies, as is the case for NODAGA-RGD: a peptide which is currently employed in clinical trials when labeled with ^68^Ga. Since application of NODAGA-RGD at later time points after injection of the radioconjugate would be of interest for nuclear physicians, we set out to investigate whether ^44^Sc-labeling with NODAGA-derivatized biomolecules is possible and whether the in vitro and in vivo behavior of the radiolabeled peptides would be equal to the DOTA-derivatized matches.

**Fig. 1:**
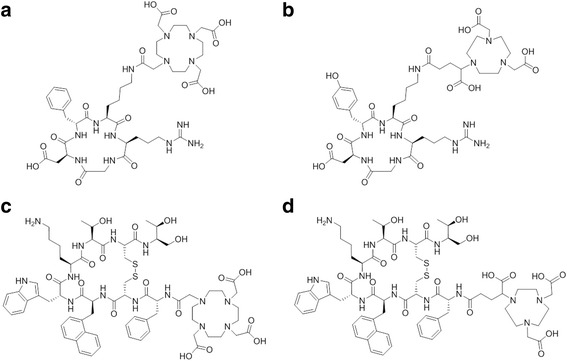
Chemical structures of DOTA-RGD (**a**), NODAGA-RGD (**b**), DOTA-NOC (**c**) and NODAGA-NOC (**d**)

The aim of this study was, therefore, to compare the in vitro and in vivo behavior of two pairs of peptides with a DOTA- and NODAGA-chelator (Fig. 1), respectively, after radiolabeling with ^44^Sc and ^68^Ga. Cyclic RGD peptides based on the Arg-Gly-Asp sequence and NOC, a somatostatin analogue ([Tyr^3^,1-NaI^3^]octreotide), were chosen as targeting agents. The in vitro stability of ^44^Sc- and ^68^Ga-labeled DOTA/NODAGA-RGD and DOTA/NODAGA-NOC was examined in saline in the presence and absence of competing metal cations. The in vivo behavior was evaluated by the performance of biodistribution studies and in vivo PET imaging of tumor-bearing mice.

## Methods

### Chemicals

^44^CaCO_3_, 97.0 % enriched (Trace Sciences International, USA) and graphite powder, 99.9999 % (Alfa Aesar, Germany) were used for target preparation. The *N,N,N’,N’*-tetra-n-octyldiglycolamide, non-branched resin (DGA, particle size 50–100 μm, TrisKem International, France) was used for the separation of Sc(III) from Ca(II). The chemical separations were performed using MilliQ water (resistance >18 MΩ) and hydrochloric acid (HCl, 30 % Suprapur, Merck KGaA, Germany). The recycling of the target material was performed with oxalic acid dihydrate, (Trace SELECT, ≥99.9999 % metals basis, Fluka Analytical, Germany) and 25 % ammonia solution (Suprapur, Merck KGaA, Germany).

^68^Ga was obtained from a ^68^Ge/^68^Ga generator IGG100 (Eckert & Ziegler, Berlin, Germany). The generator was eluted in fractions and the fraction of eluate containing the highest quantity of ^68^Ga (approximately 200–250 MBq in 700 μL 0.1 M HCl) was directly used for radiolabeling purposes without purification. DOTA-RGD (DOTA-cyclo(RGDfK) acetate, Cat-N° 9863), NODAGA-RGD (NODAGA-RGD trifluoroacetate, Cat-N° 9805), DOTA-NOC (DOTA-NOC acetate, Cat-N° 9712) and NODAGA-NOC (NODAGA-NOC acetate, Cat-N° 9718) were obtained from ABX GmbH, advanced biochemical compounds, Germany. Copper chloride dihydrate was purchased from Merck Millipore, while iron chloride hexahydrate was obtained from Sigma-Aldrich GmbH. Phosphate buffered saline (PBS) pH 7.4 was prepared in-house (Additional file 1).

### Production of ^44^Sc

^44^Sc was prepared by proton irradiation of enriched ^44^Ca targets at the Injector 2 cyclotron at PSI, as previously reported (van der Meulen et al. 2015). The irradiation of targets with 11 MeV proton beam energy, and a beam current of 50 μA, lasted for 90 min. A column (1 mL cartridge fitted with 20 μm frit ISOLUTE SPE Accessories, UK) was filled with 50 – 70 mg DGA extraction chromatographic resin and a second column with 20–25 mg of the same resin. A 20 μm frit was placed on top of the resin in each column. DGA columns were preconditioned with 3.0 M HCl. The first step of the separation was performed as previously reported (van der Meulen et al. 2015). In brief, the target was dissolved in 2.5 mL 3.0 M HCl and loaded onto the first DGA column. The rinsing of the target container and the first DGA column with 3.0 M HCl ensured a complete transfer of the ^44^Sc radioactivity and complete removal of residual Ca(II), respectively. ^44^Sc was eluted from the first DGA column with 3.5 mL 0.1 M HCl. Subsequently, the solution was acidified with the addition of 3.3 mL 6.0 M HCl to yield a 3.0 M HCl solution, which was then passed through the second DGA column, to which the ^44^Sc activity was sorbed. The elution of ^44^Sc from the second column was performed with 700 μL 0.05 M HCl (pH 1.3) and was used directly for labeling reactions. The radionuclidic purity of the ^44^Sc- eluate was quantified by γ-spectrometry using an N-type high-purity germanium (HPGe) coaxial detector (EURISYS MESURES, France) and the Ortec InterWinner 7.1 software. The ^44^Ca-contained waste fraction from the first DGA column was collected and processed to recover the target material, as described previously (van der Meulen et al. 2015).

### Radiolabeling of DOTA- and NODAGA-functionalized peptides

The total ^44^Sc and ^68^Ga activity in the obtained eluate was quantitatively determined with a dose calibrator (ISOMED 2010, Nuclear–Medizintechnik Dresden GmbH, Germany) so that the activity required for radiolabeling could be withdrawn. Sodium acetate solution (0.5 M, pH 8) was added at a ratio of 1:1 to the ^44^Sc eluate (0.05 M HCl, pH 0.4–0.6) and at a ratio of 1:2 to the ^68^Ga generator eluate (0.1 M HCl, pH 1) to give a pH of 4–4.5. The corresponding peptides (DOTA-RGD, NODAGA-RGD, DOTA-NOC and NODAGA-NOC, in a 1 mM stock solution) were added to obtain a specific activity of up to 10 MBq/nmol and the reaction mixture incubated at 95 °C for 10 min. High-performance liquid chromatography (HPLC) with a C-18 reversed–phase column (Xterra^TM^ MS, C18, 5 μm, 150 × 4.6 mm; Waters) was used for quality control. The mobile phase consisted of MilliQ water containing 0.1 % trifluoracetic acid (A) and acetonitrile (B) with a gradient of 95 % A and 5 % B to 20 % A and 80 % B over a period of 15 min at a flow rate of 1.0 mL/min.

### In vitro stability of ^44^Sc- and ^68^Ga-labeled peptides

^44^Sc- and ^68^Ga-labeled peptides (radiochemical purity >95 %) were used for the investigation of stability in 0.9 % NaCl. An activity of 80–100 MBq of ^44^Sc or ^68^Ga labeling solution was diluted with 0.9 % NaCl solution to a volume of 800 μL and incubated for four half-lives of the corresponding radionuclide at 37 °C.

Aliquots were taken from the prepared solutions at different time points over at least four half-lives, respectively, of ^44^Sc and ^68^Ga and analyzed by TLC (TLC was used, as the high metal concentrations used in this study may impair the long-term performance of HPLC columns). If initial experiments suggested instability of a compound, aliquots were retrieved more frequently. The TLC plates (Silica-gel 60, Merck) were developed using 0.1 M sodium citrate (pH 4.7) as mobile phase. The quantitative distribution of radioactivity was determined with an autoradiography system (Cyclone Plus, Perkin Elmer) and its associated software (Optiquant, version 5.0). R_f_ values of 0.2 were observed for ^44^Sc- and ^68^Ga-labeled DOTA-RGD and NODAGA-RGD, and of 0.1 for DOTA-NOC and NODAGA-NOC, respectively, whereas for unlabeled ^44^Sc and ^68^Ga a R_f_ value of 0.9 was calculated.

The influence of high metal cation concentration on the stability of ^44^Sc and ^68^Ga labeled peptides was monitored in solutions containing 0.01 M Fe^3+^ or Cu^2+^. An aqueous metal cation solution (16 μL of a 0.5 M Fe^3+^/Cu^2+^ solution) was added to 80–100 MBq of ^44^Sc or ^68^Ga labeling solution and diluted with 0.9 % NaCl solution to a volume of 800 μL. The incubation conditions, time points of retrieving samples and analysis by TLC were kept the same, as described above.

### Cell culture

U87MG cells (human glioblastoma cells; ACC® HTB-14^TM^) and AR42J cells (rat exocrine pancreatic tumor cells; ACC® CRL1492^TM^) were purchased from European Collection of Cell Cultures (ECACC, operated by Public Health England). U87MG cells were grown in MEM cell culture medium supplemented with 1 % non-essential amino acids (MEM NEAA solution 100×, Bioconcept), 1 mM sodium pyruvate (Bioconcept), 10 % fetal calf serum, L-glutamine and antibiotics. AR42J cells were grown in RPMI cell culture medium supplemented with 10 % fetal calf serum, L-glutamine and antibiotics. Routine cell culture was performed twice a week using trypsin (Gibco by life technologies 0.25 % trypsin-EDTA) for detachment of the cells.

### Tumor mouse models

In vivo experiments were approved by the local veterinarian department and conducted in accordance with the Swiss law of animal protection. Female athymic nude mice (CD-1 nude), age 5–6 weeks, were obtained from Charles River Laboratories, Sulzfeld, Germany. U87MG cells and AR42J-cells were suspended in PBS (5 × 10^6^ cells in 100 μL) and subcutaneously inoculated on each shoulder. Two to three weeks later, when the tumor reached a size of about 300–500 mm^3^, the mice were used for the in vivo studies.

### Biodistribution studies

Biodistribution studies were performed with U87MG tumor and AR42J tumor-bearing mice 2 weeks after tumor cell inoculation. ^44^Sc- and ^68^Ga-labeled peptides (~5 MBq, ~1 nmol per mouse) were intravenously injected in a volume of 100–200 μL. Mice were sacrificed at 30 min, 2 h and 5 h after injection (p.i.) of the ^44^Sc-labeled peptides. Mice which were injected with ^68^Ga-labeled peptides were sacrificed at 30 min and 2 h p.i. Selected tissues and organs were collected, weighed, and counted for radioactivity using a γ-counter (Wallac Wizard 1480, Perkin Elmer). The results were listed as a percentage of the injected activity per gram of tissue mass (% IA/g), using counts of a defined volume of the original injection solution counted at the same time. The data were analyzed for significance using a two-way ANOVA test (*Graph Pad Prism 6* software, *version 6.05*). A *p*-value of < 0.05 was considered statistically significant.

### Preclinical PET imaging

A bench-top preclinical PET scanner (G8, Sofie Biosciences, California, U.S.A. and Perkin Elmer, Massachusetts, U.S.A.) was employed for the PET scans of the tumor-bearing mice. The energy window was set to 150–650 keV. Mice were injected intravenously with ^44^Sc- or ^68^Ga-labeled peptides (~10 MBq, ~1 nmol per mouse) in a volume of 100–200 μL. The PET scans were performed 3 h and 5 h after injection of the ^44^Sc-labeled peptides and 3 h after injection of the ^68^Ga-labeled peptides, using G8 acquisition software (version 2.0.0.10). All static PET scans lasted for 20 min. During the acquisition the mice were anesthetized by inhalation of a mixture of isoflurane and oxygen. The images were reconstructed with maximum-likelihood expectation maximization (MLEM). Gauss post-reconstruction filtering was performed using VivoQuant post-processing software (version 2.10, inviCRO Imaging Services and Software, Boston, U.S.A.).

## Results

### Production and separation of ^44^Sc

^44^Sc was quantitatively sorbed on DGA resin in 3.0 M HCl solution, whereas Ca was not retained. Rinsing the resin with additional 4 mL 3.0 M HCl ensured the complete removal of Ca, after which ^44^Sc was eluted with 3.5 mL 0.1 M HCl. The ^44^Sc eluate was further acidified with the addition of 6.0 M HCl to yield a 3.0 M HCl solution. The resultant solution was passed through a second, smaller DGA column at a flow rate of ~0.3 mL/min, retaining 97 % of the eluted ^44^Sc activity. ^44^Sc was eluted quantitatively (85 ± 2 %), at activities of ~2.0 GBq, with 700 μL 0.05 M HCl and was used directly for labeling experiments. The separation procedure was initially developed using trace activities of ^46^Sc, produced by the ^45^Sc(n,γ) nuclear reaction at the BR2 reactor at SCK.CEN, Mol, Belgium.

The ^44^Sc activity separated from proton irradiated targets containing recycled ^44^CaCO_3_ was of the same quality and quantity as using the originally-purchased ^44^CaCO_3_ target material.

### Radiolabeling and stability of ^44^Sc- and ^68^Ga-labeled peptides

Radiolabeling with ^44^Sc was readily achieved with DOTA-compounds, but found to be more challenging for NODAGA-compounds, which did not allow reproducible labeling procedures at high specific activities. Radiolabeling with ^68^Ga was reproducibly achieved for both DOTA- and NODAGA-functionalized peptides, however. The radiochemical yield of the ^44^Sc and ^68^Ga radiosyntheses at the specific activity of 10 MBq/nmol was >95 %. Quantitative ^68^Ga-labeling of NODAGA-RGD and NODAGA-NOC was also possible at room temperature in less than 10 min. TLC and HPLC quality control were in good agreement. HPLC analysis demonstrated a peak of free ^44^Sc and ^68^Ga at a retention time of 2.2 ± 0.1 min, while the retention times of the radiopeptides were between 6 and 10 min (Table *1*).

**Table 1:** HPLC retention times of radiolabeled peptides

Radiopeptide	^44^Sc-/^68^Ga-DOTA-RGD	^44^Sc-/^68^Ga-NODAGA-RGD
Retention time	6.8 ± 0.1 min	6.1 ± 0.1 min
Radiopeptide	^44^Sc-/^68^Ga-DOTA-NOC	^44^Sc-/^68^Ga-NODAGA-NOC
Retention time	9.3 ± 0.1 min	9.8 ± 0.1 min

The stability of ^44^Sc- and ^68^Ga-labeled DOTA- and NODAGA-peptides was first investigated in 0.9 % NaCl over a period of four half-lives of the corresponding nuclide by means of TLC. ^44^Sc- and ^68^Ga-DOTA-RGD and DOTA-NOC exhibited a high stability. After four half-lives at 37 °C the amount of intact compound did not decrease below 98 %. ^68^Ga-labeled NODAGA-RGD and NODAGA-NOC remained stable, but the ^44^Sc-NODAGA peptides became more unstable over time. The amount of intact ^44^Sc-NODAGA-RGD dropped to 77 % and, in the case of ^44^Sc-NODAGA-NOC, a mere 37 % after more than 4 half-lives.

**Fig. 2:**
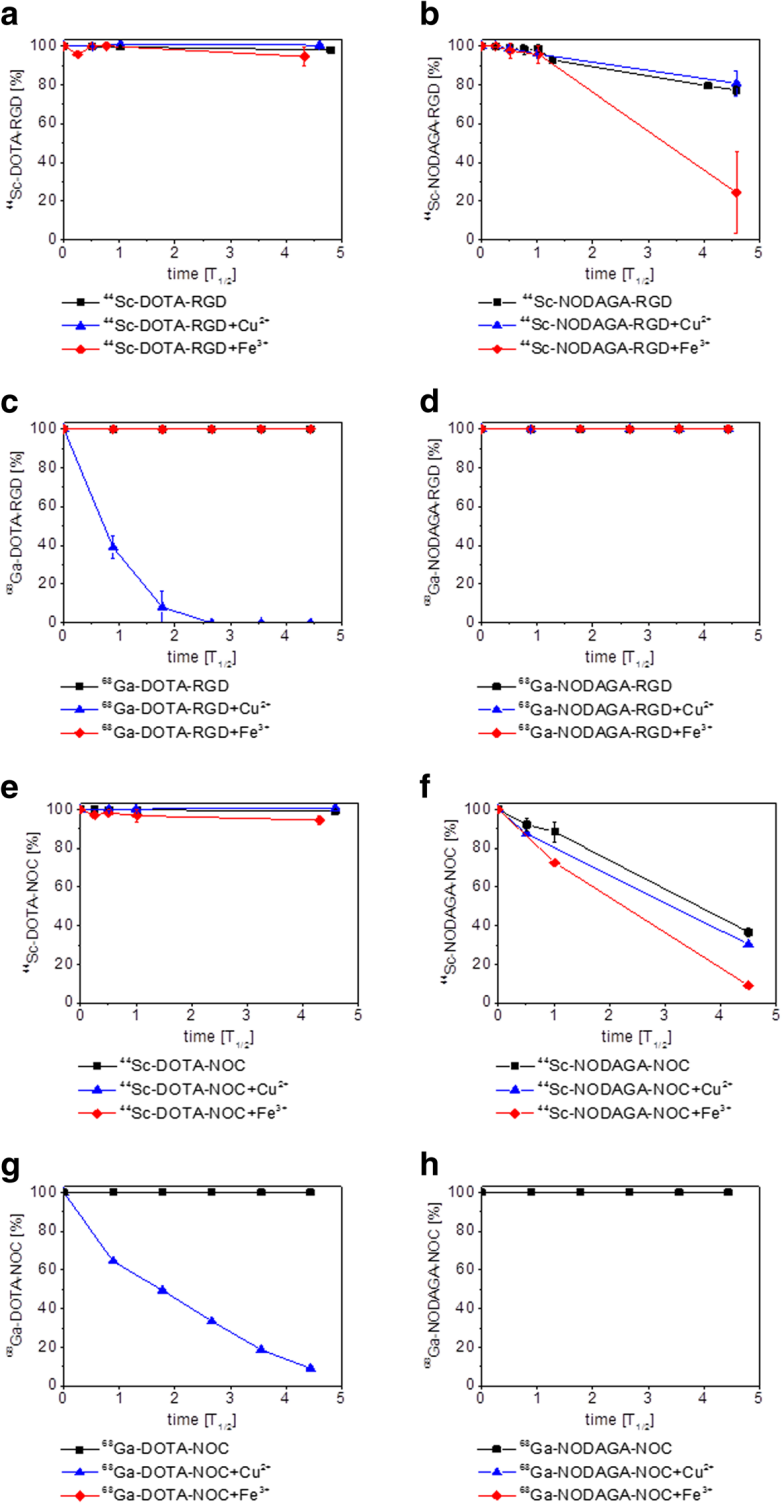
Stability of ^44^Sc-DOTA-RGD (**a**), ^44^Sc-NODAGA-RGD (**b**), ^68^Ga-DOTA-RGD (**c**), ^68^Ga-NODAGA-RGD (**d**), ^44^Sc-DOTA-NOC (**e**), ^44^Sc-NODAGA-NOC (**f**), ^68^Ga-DOTA-NOC (**g**), ^68^Ga-NODAGA-NOC (**h**) in saline with and without the presence of 10 mM Cu^2+^ and Fe^3+^, respectively

The presence of different metal cations can cause displacement of the radionuclide from the chelator and its release into solution (Pruszynski et al, 2012). The stability of ^44^Sc- and ^68^Ga-labeled DOTA- and NODAGA-peptides was investigated in the presence of Fe^3+^ and Cu^2+^ over four half-lives of the corresponding radionuclide (Fig. 2). The addition of solutions containing these metal cations to result in final metal concentrations as high as 10 mM did not induce any transmetalation of ^44^Sc-labeled DOTA- and ^68^Ga-labeled NODAGA-compounds. The integrity of ^68^Ga-DOTA-peptides was not impaired by the presence of Fe^3+^, however, the addition of 10 mM Cu^2+^ reduced the amount of intact ^68^Ga-DOTA-RGD to 10 % and ^68^Ga-DOTA-NOC to 50 %, respectively, after two half-lives. The presence of both metal cations further destabilized the already less stable ^44^Sc-labeled NODAGA-peptides.

### Biodistribution studies with ^44^Sc- and ^68^Ga-labeled peptides

Biodistribution studies were performed with ^44^Sc- and ^68^Ga-labeled DOTA/NODAGA-RGD and DOTA/NODAGA-NOC in mice bearing U87MG and AR42J tumor xenografts, respectively (Additional file 1: Table S2-S5).

**Fig. 3:**
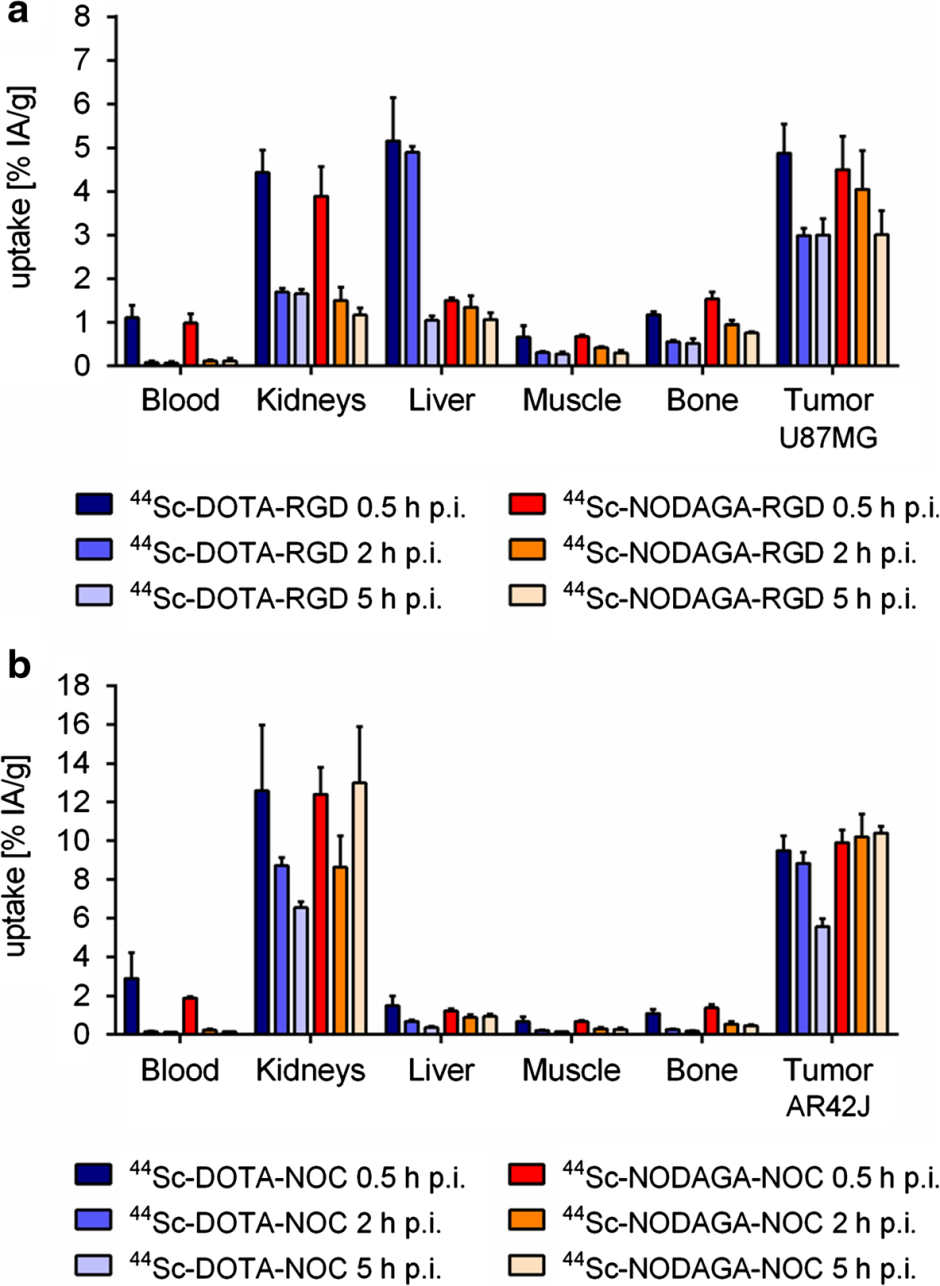
Biodistribution data obtained at different time points after injection of *~*5 MBq (1 nmol) ^44^Sc-DOTA/NODAGA-RGD in U87MG tumor-bearing mice (**a**) and after injection of *~*5 MBq (1 nmol) ^44^Sc-DOTA/NODAGA-NOC in AR42J tumor-bearing mice (**b**), respectively. Data bars represent the average ± SD of values obtained from *n* = 3 mice

Time-dependent distribution studies of ^44^Sc-DOTA-RGD and ^44^Sc-NODAGA-RGD revealed a similar pattern for both compounds, resulting in a tumor uptake of 4.88 ± 0.67 % IA/g and 4.50 ± 0.77 % IA/g, respectively, at 0.5 h after injection (Fig. 3a). The wash-out of radioactivity from the tumor tissue was somewhat faster for the ^44^Sc-DOTA-RGD than for the ^44^Sc-NODAGA-RGD, but at 5 h after injection the values were almost the same (3.00 ± 0.38 % IA/g vs 3.01 ± 0.55 % IA/g). Clearance from the blood was fast for both ^44^Sc-DOTA-RGD and ^44^Sc-NODAGA-RGD. This was also reflected by the high renal uptake shortly after injection (4.44 ± 0.51 % IA/g vs 3.89 ± 0.68 % IA/g, 0.5 h p.i.) which decreased with time, resulting in a retention of <2 % IA/g at 5 h p.i. Whereas the accumulation in non-targeted organs and tissues was generally comparable, the liver uptake of ^44^Sc-DOTA-RGD (5.16 ± 0.99 % IA/g, 0.5 h) was clearly higher than for ^44^Sc-NODAGA-RGD (1.49 ± 0.06 % IA/g, 0.5 h) at early time points. At 5 h after injection uptake in the liver was ~1 % IA/g for both radiopeptides (Fig. 3a).

The tissue distribution pattern of ^44^Sc-DOTA-NOC and ^44^Sc-NODAGA-NOC was comparable with regard to the uptake in AR42J tumors (9.49 ± 0.76 % IA/g vs. 9.90 ± 0.66 % IA/g), kidneys (12.6 ± 3.36 % IA/g vs. 12.4 ± 1.41 % IA/g) and liver (1.49 ± 0.49 % IA/g vs. 1.22 ± 0.11 % IA/g) at 0.5 h after injection (Fig. 3b). The tissue distribution profiles at 2 h after injection of the NOC-based radiopeptides were also comparable. At 5 h after injection, retention of ^44^Sc-DOTA-NOC in tumors (5.56 ± 0.40 % IA/g) was lower than for ^44^Sc-NODAGA-NOC (10.8 ± 0.37 % IA/g), which indicates a faster wash-out of ^44^Sc-DOTA-NOC. Renal retention of ^44^Sc-DOTA-NOC decreased further over the period of investigation (6.54 ± 0.30 % IA/g, 5 h p.i.) while the accumulation of activity in the kidneys (13.0 ± 2.98 % IA/g) was increased at 5 h after injection of ^44^Sc-NODAGA-NOC (Fig. 3b).

**Fig. 4:**
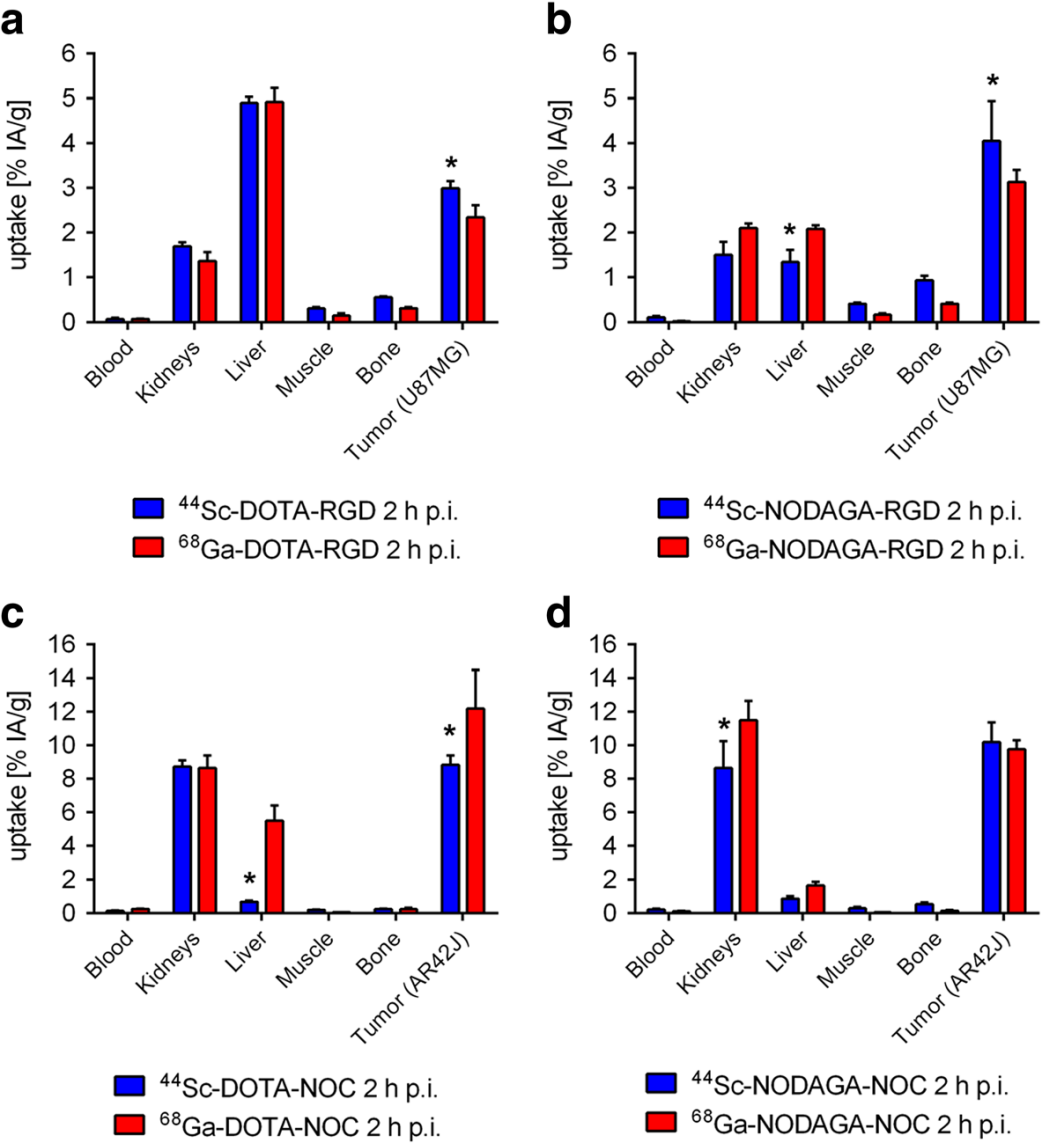
Biodistribution data obtained 2 h after injection of *~*5 MBq (*~*1 nmol) ^44^Sc- and ^68^Ga-labeled DOTA-RGD (**a**) and NODAGA-RGD (**b**) and *~*5 MBq (*~*1 nmol) DOTA-NOC (**c**) and NODAGA-NOC (**d**). Data bars represent the average ± SD of values obtained from *n* = 3 mice (* significantly different uptake of the ^44^Sc-labeled peptide compared to the ^68^Ga-labeled peptide, *p <*0.05)

For selected organs and tissues, the uptake of the ^44^Sc-labeled peptides was compared with the uptake of the ^68^Ga-labeled peptides in mice at 2 h after injection (Fig. 4). The tissue distribution of DOTA-RGD was almost the same, independent of the radionuclide (^44^Sc vs ^68^Ga) used (Fig. 4a), although the tumor uptake was higher for ^44^Sc-DOTA-RGD (2.99 ± 0.16 % IA/g) than for ^68^Ga-DOTA-RGD (2.35 ± 0.27 % IA/g, *p* <0.05). The NODAGA-derivatized RGD-peptides revealed the same trend: the tumor uptake of ^44^Sc-NODAGA-RGD (4.05 ± 0.89 IA/g) was significantly higher (*p* <0.05) than the tumor uptake of ^68^Ga-NODAGA-RGD (3.13 ± 0.27 % IA/g). Undesired accumulation of ^44^Sc-NODAGA-RGD in the liver (1.34 ± 0.27 % IA/g, *p* <0.05) was significantly reduced compared to ^68^Ga-NODAGA-RGD (2.09 ± 0.08 % IA/g, Fig. 4b). Renal uptake of ^44^Sc-NODAGA-RGD was also slightly lower than for ^68^Ga-NODAGA-RGD. The DOTA-RGD accumulated to a higher extent in the liver than the NODAGA-RGD, irrespective of which radionuclide was used for labeling (Fig. 4a/b). In all other organs of interest, the distribution profile was roughly the same, irrespective of the chelator used for coordination of the radionuclide.

Accumulation of ^44^Sc-DOTA-NOC in the tumor xenografts (8.83 ± 0.57 % IA/g) was significantly lower (*p* <0.05) when compared to the uptake of ^68^Ga-DOTA-NOC (12.2 ± 2.29 % IA/g) (Fig. 4c). This was also the case in the liver, where the uptake of ^44^Sc-DOTA-NOC (0.68 ± 0.07 % IA/g) was significantly (*p* <0.05) lower than for ^68^Ga-DOTA-NOC (5.52 ± 0.88 % IA/g). In all other organs and tissues the distribution of radioactivity was comparable among the radiopeptides, irrespective of whether they were labeled with ^44^Sc or ^68^Ga (Fig. 4c). The tissue distribution of ^44^Sc-NODAGA-NOC and ^68^Ga-NODAGA-NOC was also comparable (Fig. 4d). The only significant difference (*p* <0.05) were the kidneys, in which ^44^Sc-NODAGA-NOC was less retained (8.64 ± 1.62 % IA/g) than the ^68^Ga-NODAGA-NOC (11.5 ± 1.15 % IA/g). The liver uptake of ^44^Sc-NODAGA-NOC (0.88 ± 0.15 % IA/g) was lower than for ^68^Ga-NODAGA-NOC (1.68 ± 0.19 % IA/g) but the difference was not significant (Fig. 4c). Comparison of the DOTA-NOC and NODAGA-NOC revealed similar distribution profiles, irrespective of the radionuclide employed. As the only exception, it should be mentioned that ^68^Ga-DOTA-NOC showed a clearly higher retention in the liver than all other NOC-based radiopeptides (Fig. 4c/d).

As a result of the tissue distribution data reported above, the tumor-to-background ratios were mostly similar between ^44^Sc-labeled DOTA-RGD and NODAGA-RGD as well as between ^44^Sc-labeled DOTA-NOC and NODAGA-NOC, respectively (Tables 2 and 3). When comparing variation of tumor-to-background ratios between the ^44^Sc- and ^68^Ga-labeled versions of each of the four peptides, the ratios appeared more pronounced for the NOC-based radiopeptides over their RGD-based counterparts (Tables 2 and 3).

**Table 2:** Tumor-to-background ratios at different time points after injection of ^44^Sc/^68^Ga-labeled DOTA-RGD and NODAGA-RGD

	^44^Sc-DOTA-RGD			^68^Ga-DOTA-RGD	
U87MG	30 min p.i.	2 h p.i.	5 h p.i.	30 min p.i.	2 h p.i.
Tumor-to-blood	4.82 ± 1.60	40.1 ± 15.6	47.2 ± 18.0	4.34 ± 0.57	28.0 ± 4.26
Tumor-to-liver	0.99 ± 0.14	0.61 ± 0.04	2.90 ± 0.38	0.55 ± 0.09	0.48 ± 0.07
Tumor-to-kidney	1.16 ± 0.29	1.77 ± 0.13	1.82 ± 0.21	0.88 ± 0.07	1.74 ± 0.19
	^44^Sc-NODAGA-RGD			^68^Ga-NODAGA-RGD	
U87MG	30 min p.i.	2 h p.i.	5 h p.i.	30 min p.i.	2 h p.i.
Tumor-to-blood	4.73 ± 1.24	34.1 ± 7.62	30.5 ± 9.93	4.17 ± 0.47	114 ± 35.0
Tumor-to-liver	3.02 ± 0.40	3.01 ± 0.13	2.82 ± 0.21	1.72 ± 0.16	1.50 ± 0.20
Tumor-to-kidney	1.18 ± 0.28	2.68 ± 0.14	2.58 ± 0.24	0.89 ± 0.03	1.49 ± 0.21

**Table 3:** Tumor-to-background ratios at different time points after injection of ^44^Sc/^68^Ga-labeled DOTA-NOC and NODAGA-NOC

	^44^Sc-DOTA-NOC			^68^Ga-DOTA-NOC	
AR42J	30 min p.i.	2 h p.i.	5 h p.i.	30 min p.i.	2 h p.i.
Tumor-to-blood	3.69 ± 1.52	58.2 ± 6.64	52.0 ± 4.38	5.22 ± 1.07	46.2 ± 2.28
Tumor-to-liver	6.87 ± 2.38	13.1 ± 2.16	15.7 ± 2.82	2.44 ± 0.36	2.22 ± 0.22
Tumor-to-kidney	0.79 ± 0.19	1.01 ± 0.06	0.85 ± 0.04	1.20 ± 0.21	1.41 ± 0.18
	^44^Sc-NODAGA-NOC			^68^Ga-NODAGA-NOC	
AR42J	30 min p.i.	2 h p.i.	5 h p.i.	30 min p.i.	2 h p.i.
Tumor-to-blood	5.23 ± 0.22	50.3 ± 16.8	78.6 ± 6.95	4.32 ± 0.58	76.0 ± 4.31
Tumor-to-liver	8.13 ± 0.72	11.9 ± 3.26	11.6 ± 1.51	4.36 ± 0.91	5.88 ± 0.68
Tumor-to-kidney	0.80 ± 0.10	1.22 ± 0.34	0.87 ± 0.24	0.58 ± 0.10	0.85 ± 0.08

### Preclinical PET imaging studies with ^44^Sc- and ^68^Ga-labeled peptides

**Fig. 5:**
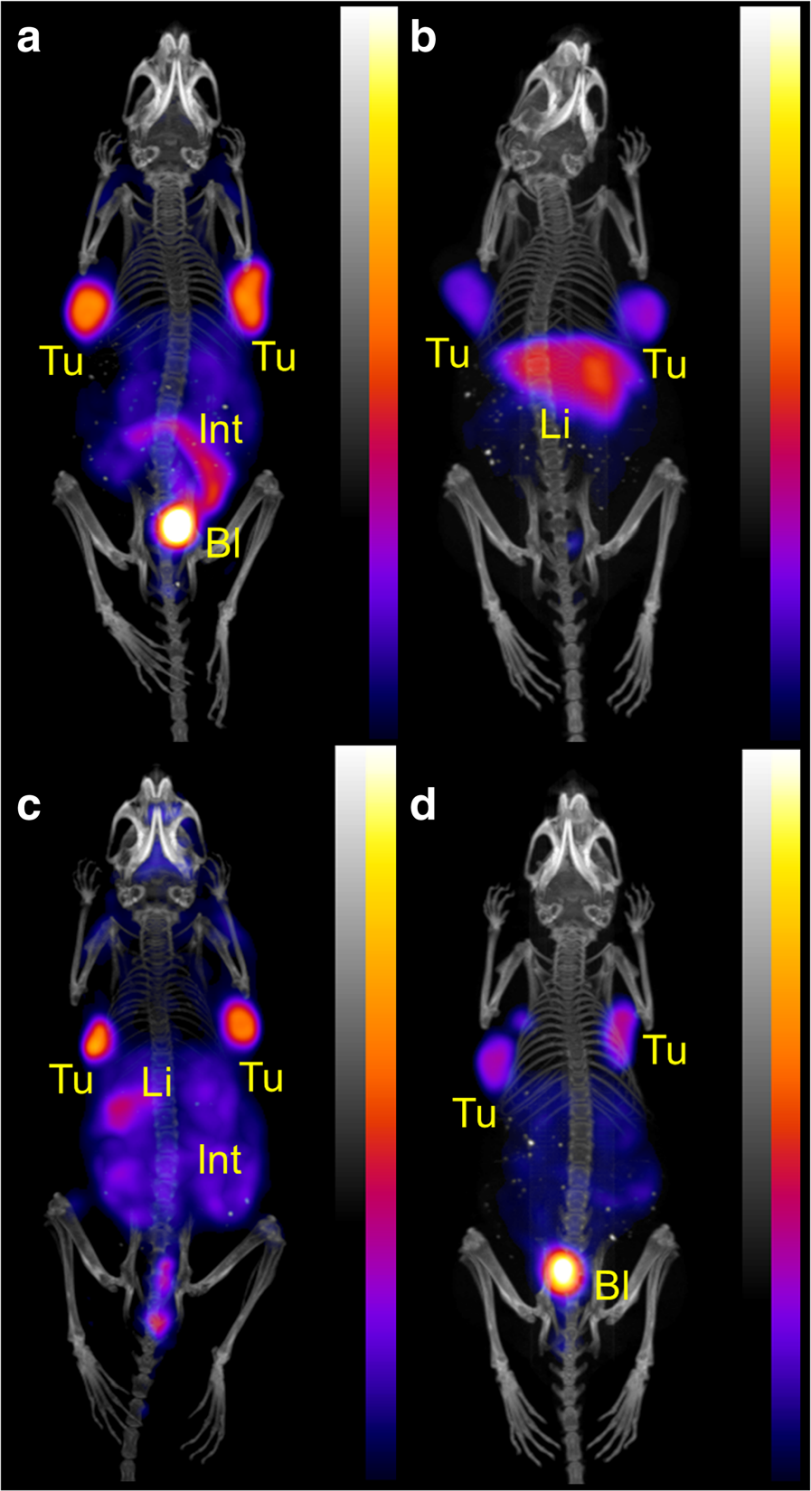
PET/CT scans of U87MG tumor-bearing mice 3 h after injection of ~10 MBq (~1 nmol) ^44^Sc-DOTA-RGD (**a**), (~10 MBq/~1 nmol) ^68^Ga-DOTA-RGD (**b**), ~10 MBq (~1 nmol) ^44^Sc-NODAGA-RGD (**c**) and ~10 MBq (~1 nmol) ^68^Ga-NODAGA-RGD (**d**). During the PET (20 min) and the CT (1.5 min) scans the mice were anesthetized with isoflurane/oxygen (Tu = U87MG tumor xenografts, Li = liver, Int = intestines, Bl = urinary bladder)

**Fig. 6:**
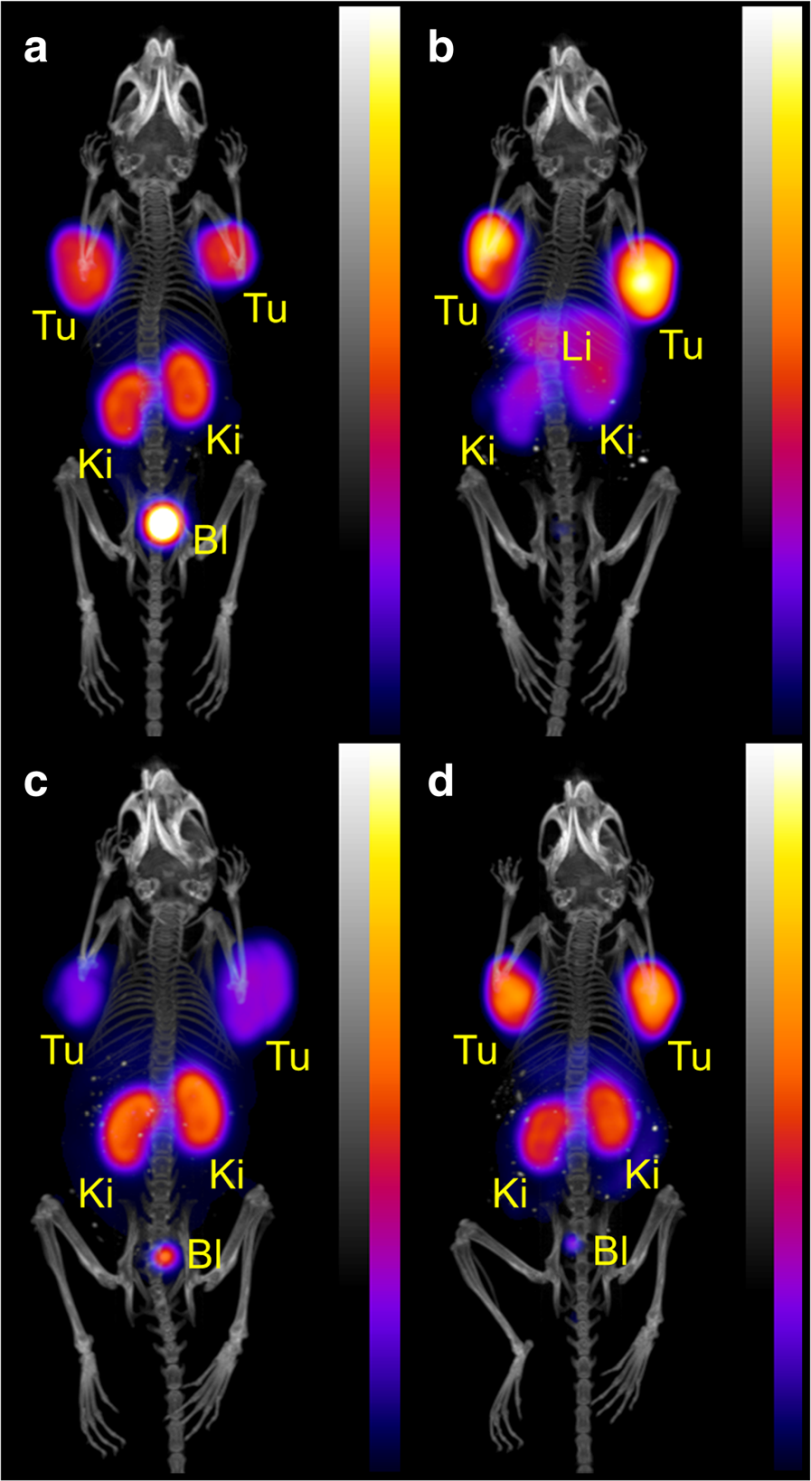
PET/CT scans of AR42J tumor-bearing mice 3 h after injection of ~10 MBq (~1 nmol) ^44^Sc-DOTA-NOC (**a**), ~10 MBq (~1 nmol) ^68^Ga-DOTA-NOC (**b**), ~10 MBq (~3 nmol) ^44^Sc-NODAGA-NOC (**c**) and ~10 MBq (~1 nmol) ^68^Ga-NODAGA-NOC (**d**). During the PET (20 min) and the CT (1.5 min) scans the mice were anesthetized with isoflurane/oxygen (Tu = U87MG tumor xenografts, Li = liver, Int = intestines, Bl = urinary bladder)

PET/CT experiments were performed with one or two mice 3 h after injection of ^44^Sc- and ^68^Ga-labeled DOTA-RGD, NODAGA-RGD, DOTA-NOC and NODAGA-NOC, respectively (Figs. 5 and 6). ^44^Sc-DOTA-RGD showed clearly less accumulation of radioactivity in the liver than ^68^Ga-DOTA-RGD (Fig. 5a/b). ^44^Sc-NODAGA-RGD was comparable to ^68^Ga-NODAGA-RGD but showed somewhat more background activity in the abdominal tract (Fig. 5c/d). The uptake pattern of ^44^Sc-DOTA-RGD was slightly more favorable over the distribution of ^44^Sc-NODAGA-RGD (Fig. 5a/c).

PET/CT scans obtained at 3 h after injection of ^44^Sc-DOTA-NOC revealed uptake of radioactivity only in the tumor xenografts and in the kidneys, while ^68^Ga-DOTA-NOC accumulated also to a significant extent in the liver (Fig. 6a/b). ^44^Sc-NODAGA-NOC and ^68^Ga-NODAGA-NOC accumulated solely in tumors and kidneys (Fig. 6c/d). The tumor uptake of ^44^Sc-NODAGA-NOC in the mouse, which was used for PET imaging, was reduced, resulting in lower tumor-to-kidney ratios compared to ^68^Ga-NODAGA-NOC. In this context, it has to be mentioned that ^44^Sc-NODAGA-NOC was prepared at a low specific activity for the PET scan (Fig. 6c) which implies that the injected molar amount of peptide was significantly increased compared to the peptide amount injected with ^68^Ga-NODAGA-NOC and, as a result, the binding sites in the tumor tissue may have been saturated.

**Fig. 7:**
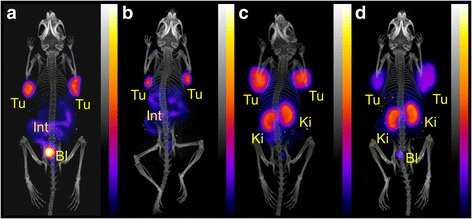
PET/CT scans of U87MG tumor-bearing mice 5 h after injection of ~10 MBq (~1 nmol) ^44^Sc-DOTA-RGD (**a**) and ~10 MBq (~1 nmol) ^44^Sc-NODAGA-RGD (**b**). PET/CT scans of AR42J tumor-bearing mice 5 h after injection of ~10 MBq (~1 nmol) ^44^Sc-DOTA-NOC (**c**) and ~10 MBq (~3 nmol) ^44^Sc-NODAGA-NOC (**d**). During the PET (20 min) and the CT (1.5 min) scans the mice were anesthetized with isoflurane/oxygen (Tu = U87MG (**a/b**) or AR42J tumor xenografts (**c/d**), Ki = kidney, Int = intestines, Bl = urinary bladder)

An overview of the PET/CT scans of all four peptides labeled with ^44^Sc at 5 h after injection showed largely the same tissue distribution as was found at 3 h after injection (Fig. 7). While RGD-based peptides accumulated in the U87MG tumor xenografts and showed residual activity in the intestinal tract, NOC-based peptides accumulated in AR42J tumors xenografts and showed significant retention of radioactivity in the kidneys.

## Discussion

A number of preclinical studies demonstrated the potential of ^44^Sc as an alternative PET radiometal to the currently-used ^68^Ga (Müller et al. 2013; Koumarianou et al. 2012; Hernandez et al. 2014; Miederer et al. 2011). With this in mind, the possibility to extend its applications to peptides of clinical relevance is of great interest for medical physicians. Herein, we reported on the first, to our knowledge, preclinical study concerning the ^44^Sc-labeling of peptides comprising a NODAGA-chelator, as well as their in vitro and in vivo behavior. Several authors proposed the DOTA-chelator as the most suitable ligand for binding Sc(III), whereas for Ga(III) it is known that NODAGA complexes provide higher thermodynamic stability than the DOTA complex (Huclier-Markai et al. 2011; Notni et al. 2012). Currently, the ^68^Ga-DOTA functionalized somatostatin receptor analogues are among the most prominent radiopharmaceuticals for clinical PET imaging (Banerjee & Pomper 2013). Since NODAGA derivatized biomolecules have not been used for labeling with ^44^Sc to date, the question arose on whether or not sufficient stability can be achieved for in vivo application of ^44^Sc-NODAGA compounds.

In order to perform the preclinical experiments effectively, it was necessary to obtain the ^44^Sc in a small solution volume, suitable for direct radiolabeling and subsequent in vivo application, without extensive dilution. Previously, we reported on the implementation of SCX resin to concentrate the ^44^Sc radioactivity in a small volume (van der Meulen et al. 2015). Although the use of this resin is already established for the concentration of the ^68^Ga eluate from the ^68^Ge generator, the high osmolarity of the eluate is not suitable for direct in vivo application. Herein, we report the use of DGA extraction chromatographic resin to effectively concentrate ~85 % of the ^44^Sc radioactivity in a small volume of 700 μL. The acidic solution containing the ^44^Sc was mixed with sodium acetate to obtain a pH of 4–4.5 for radiolabeling reactions. This procedure allowed in vivo application of the radiolabeled peptides without excessive dilution.

Reproducible labeling with ^44^Sc at high specific activities (10 MBq/nmol) was achieved for peptides functionalized with a DOTA-chelator. The same was possible for ^68^Ga with both DOTA- and NODAGA-functionalized peptides. Radiolabeling of NODAGA-compounds with ^44^Sc, however, proved to be more challenging and was not achieved reproducibly at high specific activity. It may be a result of potentially interfering metal contaminations to which NOTA/NODAGA is more susceptible than DOTA, as previously reported (Simecek et al. 2013). If NODAGA-functionalized peptides should be used with ^44^Sc for clinical studies, it will be important to determine the maximum concentration of metal contaminants which would still allow high specific and reproducible labeling with ^44^Sc. A potential optimization of the labeling may also be accessible by thorough investigation of different buffer systems and the use of microwave heating (Elander et al. 2000). Finally, even when the labeling was achieved successfully, the stability of ^44^Sc-labeled NODAGA-peptides was clearly inferior to the stability of ^44^Sc-labeled DOTA-compounds. It will be important, thus, to investigate the conditions which enhance the stability of ^44^Sc-NODAGA-peptides, potentially allowing an increased shelf-life which would be necessary in view of a clinical application.

In the initial in vitro test, the stability of ^44^Sc- and ^68^Ga-labeled peptides was investigated in saline with and without addition of excess and Fe^3+^ and Cu^2+^, respectively, to determine the possibility of metal challenge. None of the conditions impaired the integrity of ^44^Sc-labeled DOTA-RGD and DOTA-NOC, even after four half-lives of incubation at 37 °C. The obtained results are in agreement with those of Pruszynski et al., who reported an unchanged stability of ^44^Sc-DOTA-TOC in the presence of metal cations (Pruszynski et al. 2012). The amount of intact ^68^Ga-DOTA-RGD and ^68^Ga-DOTA-NOC was only decreased after the addition of Cu^2+^ (0.01 M) which was comparable with the time- and Cu^2+^-concentration dependent transmetalation of ^68^Ga-DOTA-TATE (Oehlke et al. 2013). ^44^Sc-labeled NODAGA-peptides were significantly less stable, indicating an onset of release of the radionuclide from the chelator only one half-life after labeling. This was in clear contrast to the ^68^Ga-NODAGA-peptides, which were completely stable over the whole period of investigation. The NODAGA-chelator revealed less stable coordination of ^44^Sc compared to the DOTA under the experimental conditions in this work. Distribution coefficients determined in n-octanol and PBS pH 7.4 revealed logD values in the same range (−4.70 to −4.26) for all RGD-based peptides, irrespective of the chelator and radionuclide which was employed. The logD values obtained with NOC-based peptides were slightly higher (−1.68 to −2.54) for all four radiopeptides (^44^Sc/^68^Ga-DOTA/NODAGA-NOC), indicating increased lipophilic properties compared to the RGD-peptides (Additional file 1: Table S1).

Biodistribution studies were performed with tumor-bearing mice at different time points after administration of the ^44^Sc-labeled peptides. Application of ^44^Sc-DOTA/NODAGA-RGD and ^44^Sc-DOTA/NODAGA-NOC, respectively, resulted in only small variations of the kinetics, independent of whether a DOTA or a NODAGA chelator was used (Fig. 3b). Comparing the tissue distribution profiles of ^44^Sc-labeled peptides with those of ^68^Ga-labeled peptides revealed that the differences between DOTA- and NODAGA-derivatized compounds were largely due to the different properties of these peptides, rather than the consequence of any kind of instability (Fig. 4). Indications of in vivo instability of the ^44^Sc-NODAGA-compounds were not apparent, with the exception of the increasing kidney uptake from 3 to 5 h after injection of ^44^Sc-NODAGA-NOC (Fig. 3b). It remained unclear, however, whether this was due to release of ^44^Sc from the NODAGA chelator since injection of free ^44^Sc resulted in unspecific retention of radioactivity in the liver and intestinal tract, rather than in the kidneys (Additional file 1: Figure S1). Generally, the instability of ^44^Sc-NODAGA compounds in solution was time-dependent and, as a consequence, it appeared not to be an issue for imaging purposes at relatively short time points (<1 half-life of ^44^Sc) after injection. The tissue distribution profiles with each peptide were similar, independent of whether it was labeled with ^44^Sc or ^68^Ga. One of the most conspicuous differences between ^44^Sc- and ^68^Ga-labeled peptides, however, was the increased liver uptake of ^68^Ga-DOTA-peptides, which was seen in PET images obtained with both DOTA-RGD and DOTA-NOC, respectively. Biodistribution studies confirmed these differences in liver uptake for DOTA-NOC (Fig. 4c), however, in the case of DOTA-RGD the liver uptake was relatively high for both ^68^Ga-DOTA-RGD and ^44^Sc-DOTA-RGD (Fig. 4a). This was in contrast to ^44^Sc- and ^68^Ga-labeled NODAGA-RGD peptides, which showed a clearly reduced liver uptake at 2 h p.i. in comparison (Fig. 4b). Since uncoordinated ^68^Ga may also accumulate to a significant extent in the bones as it was shown in a separate experiment (Additional file 1: Figure S1), it is unlikely that the described results are a consequence of released ^68^Ga from the DOTA-chelator.

Previously, it was reported that ^68^Ga-DOTA-RGD showed a higher blood pool activity than ^68^Ga-NODAGA-RGD (Knetsch et al. 2011; Decristoforo et al. 2008). As a result and in agreement with our studies, it was found that ^68^Ga-DOTA-RGD accumulates to a higher extent in the liver than ^68^Ga-NODAGA-RGD (Fig. 4a/b). In this context, it is also important to note that the peptide structure of the DOTA-RGD comprises a phenylalanine, whereas in the case of the NODAGA-RGD the phenylalanine was replaced with a tyrosine, which may have an influence on the pharmacokinetics of these peptides (Fig. 1). Overall, ^68^Ga-NODAGA-RGD revealed a more favorable tissue distribution profile than ^68^Ga-DOTA-RGD, shown in previous studies as well as in the experiments presented in this work (Knetsch et al. 2011; Pohle et al. 2012). A similar trend, albeit less pronounced, was seen for the ^44^Sc-labeled RGD peptides, showing lower background activity of ^44^Sc-NODAGA-RGD compared to that of ^44^Sc-DOTA-RGD (Fig. 4a/b).

The most striking difference between ^44^Sc- and ^68^Ga-labeled NOC-based peptides was the reduced liver uptake of ^44^Sc-DOTA-NOC as compared to ^68^Ga-DOTA-NOC, although ^68^Ga-DOTA-NOC accumulated to a higher extent in the tumor tissue than ^44^Sc-DOTA-NOC (Fig. 4c). When looking at the tissue distribution profiles of the ^44^Sc- and ^68^Ga-NODAGA-NOC peptides, they were found to be largely comparable with slightly higher retention of ^68^Ga-NODAGA-NOC in the kidneys (Fig. 4d). These results were also largely comparable to previously published data obtained with ^68^Ga-NODAGA-TOC (Eisenwiener et al. 2002). Even though the ^44^Sc-NODAGA-NOC revealed to be the least stable in vitro, its tissue distribution profile was largely comparable to ^68^Ga-labeled NODAGA-NOC indicating that the compound was stable in vivo.

Overall, it was found that ^44^Sc-DOTA-peptides were of significantly higher stability than the corresponding ^44^Sc-NODAGA-peptides, as expected, based on stability constants previously reported (Huclier-Markai et al. 2011; Port et al. 2008). Our experience also revealed that metal impurities would clearly interfere more distinctly with the stability of ^44^Sc-NODAGA-compounds than in the case of ^44^Sc-DOTA-compounds. Finally, it is important to note that the ^44^Sc-labeling of a NODAGA-functionalized biomolecule appears to be dependent on the overall chemical structure of the compound, as the ^44^Sc-NODAGA-RGD was found to be clearly more stable than the ^44^Sc-NODAGA-NOC. NODAGA-chelators are, thus, not excluded from use for labeling with ^44^Sc, but a thorough investigation of each case will be necessary in order to guarantee sufficient stability of the radiopharmaceutical.

Based on our results, which show clear differences in kinetics between labeled DOTA- and NODAGA-functionalized peptides, it is likely that ^44^Sc would be the preferred nuclide to be used with DOTA-functionalized biomolecules, in order to reflect the tissue distribution of ^177^Lu-labeled compounds more accurately, than if ^68^Ga was used for the same purpose.

## Conclusions

In this work, it was demonstrated that ^44^Sc can be used for the labeling of biomolecules with both a DOTA and NODAGA chelator, although using a NODAGA-chelator proved to be more challenging. Other than with ^68^Ga, which shows clearly better results if coordinated with a NODAGA-chelator, ^44^Sc appears to be more stably complexed with DOTA. Based on these results, we conclude that even though ^44^Sc would be most favorably coordinated with a DOTA-chelator, coordination with a NODAGA-chelator is possible if the labeling conditions and storage buffers are validated. When using ^44^Sc and ^47^Sc for theragnostic application, however, DOTA is clearly the chelator of choice.

### Compliance with Ethical Standards

#### Ethical approval

All applicable international, national, and/or institutional guidelines for the care and use of animals were followed.

#### Ethical approval

This article does not contain any studies with human participants performed by any of the authors.

## Additional file

Additional file 1: Supplementary experimental data. (DOCX 777 kb)
