# Supplementary material for: 44Sc for labeling of DOTA- and NODAGA-functionalized peptides: preclinical in vitro and in vivo investigations
Source: EJNMMI Radiopharm Chem. 2016 May 5;1:8. doi: 10.1186/s41181-016-0013-5 (PMC5843811; doi:10.1186/s41181-016-0013-5)
Supplement: Supplementary file 1 — Supplementary experimental data. (DOCX 777 kb) [file 41181_2016_13_MOESM1_ESM.docx]

Additional file 1

**^44^Sc for labeling of DOTA- and NODAGA-functionalized peptides: preclinical in vitro and in vivo investigations**

Katharina A. Domnanich^1,2^, Cristina Müller^3,4^, Renata Farkas^3^, Raffaella M. Schmid^3^,. Bernard Ponsard^5^, Roger Schibli^3,4^_,_ Andreas Türler^1,2^, Nicholas P. van der Meulen^1,3*^

*^1^Laboratory of Radiochemistry, Paul Scherrer Institute, 5232 Villigen-PSI, Switzerland*

*^2^Department of Chemistry and Biochemistry, University of Bern, 3012 Bern, Switzerland*

*^3^Center for Radiopharmaceutical Sciences ETH-PSI-USZ, Paul Scherrer Institute, 5232 Villigen-PSI, Switzerland*

*^4^Department of Chemistry and Applied Biosciences, ETH Zurich, 8093 Zurich, Switzerland*

*^5^SCK.CEN, BR2 Reactor, 2400 Mol, Belgium*

Email-addresses:

[katharina.domnanich@psi.ch](mailto:katharina.domnanich@psi.ch); [cristina.mueller@psi.ch](mailto:cristina.mueller@psi.ch); [renata.farkas@psi.ch](mailto:renata.farkas@psi.ch); [raffaella.schmid@psi.ch](mailto:raffaella.schmid@psi.ch); [bernard.ponsard@sckcen.be](mailto:bernard.ponsard@sckcen.be); [roger.schibli@psi.ch](mailto:roger.schibli@psi.ch); [andreas.tuerler@psi.ch](mailto:andreas.tuerler@psi.ch); [nick.vandermeulen@psi.ch](mailto:nick.vandermeulen@psi.ch)

*** Corresponding author:**

Dr. Nicholas P. van der Meulen

Laboratory of Radiochemistry

Paul Scherrer Institute

CH-5232 Villigen-PSI

Switzerland

e-mail: nick.vandermeulen@psi.ch

phone: +41-56-310 50 87

fax: +41-56-310 28 49

**Preparation of Phosphate Buffered Saline (PBS) pH 7.4**

For the preparation of in-house used PBS, sodium chloride (2.9 g NaCl) and sodium phosphate salts (3.0 g Na_2_HPO_4_ x H_2_O and 0.75 g NaH_2_PO_4_ x H_2_O) were dissolved in 800 mL MilliQ water. The pH value was adjusted to 7.4 by dropwise addition of 5 N NaOH followed by adjustment of the volume to 1.0 L using MilliQ water. Before use, the PBS pH 7.4 solution was filtered using a 0.2 μm filter.

**Determination of distribution coefficients (logD values)**

*Experimental:* The distribution coefficient (logD) of all four derivatives was determined using a previously published procedure [1]. In brief, a sample of ~2.5 MBq of ^68^Ga labeled compound or a sample of ~1.25 MBq of ^44^Sc labeled compound was mixed with an equal volume of PBS (pH 7.4) and *n-*octanol (1500 μL). The vials were vortexed vigorously for 1 min and then centrifuged for 6 min to achieve phase separation. The concentration of radioactivity in a defined volume of each layer was measured in a γ-counter (Wallac Wizard 1480, Perkin Elmer). The distribution coefficient was expressed as the logarithm of the ratio of counts per minute (cpm) measured in the *n-*octanol phase to the cpm measured in the PBS phase. The values reported are the mean of two to three independent measurements (± SD), each performed with five replicates.

*Results:* The logD values obtained from the distribution of the radiolabeled peptides between *n-*octanol and PBS pH 7.4 were determined for each peptide. For DOTA-RGD and DOTA-NOC, the logD values were similar for the ^44^Sc- and ^68^Ga-labeled peptides. The NODAGA-functionalized peptides showed significantly lower values for the ^44^Sc-labeled peptides than for the ^68^Ga-labeled peptides, however. The comparison of ^44^Sc-DOTA-RGD with ^44^Sc-NODAGA-RGD and ^44^Sc-DOTA-NOC with ^44^Sc-NODAGA-NOC revealed similar logD values independent on the chelator which was employed (Supplementary Table S1). The values of the ^44^Sc labeled DOTA-peptides were well comparable to the values obtained with the ^68^Ga-labeled DOTA-peptides. Evaluation of the ^44^Sc-NODAGA-peptides revealed lower values than the ^68^Ga labeled counterparts (Supplementary Table S1).

**Supplementary Table S1** Distribution coefficients (logD values) of each peptide labeled with either ^44^Sc or ^68^Ga.

| Radionuclide | DOTA-RGD | NODAGA-RGD | DOTA-NOC | NODAGA-NOC |
| --- | --- | --- | --- | --- |
| ^44^Sc | -4.48 ± 0.04 | -4.70 ± 0.18 | -2.54 ± 0.31 | -2.35 ± 0.06 |
| ^68^Ga | -4.59 ± 0.06 | -4.26 ± 0.01 | -2.26 ± 0.05 | -1.68 ± 0.07 |

**Biodistribution Data**

Biodistribution studies were performed with ^44^Sc and ^68^Ga labeled DOTA-RGD and NODAGA-RGD, respectively, in U87MG tumor-bearing mice (Supplementary Tables S2/S3).

**Supplementary Table S2.** Biodistribution of ^44^Sc-DOTA-RGD and ^68^Ga-DOTA-RGD in U87MG tumor-bearing female nude mice, expressed in percentage of total injected activity per gram tissue (% IA/g)

|  | ^44^Sc-DOTA-RGD | | | ^68^Ga-DOTA-RGD | |
| --- | --- | --- | --- | --- | --- |
|  | 30 min p.i. | 2 h p.i. | 5 h p.i. | 30 min p.i. | 2 h p.i. |
| Blood | 1.10 ± 0.29 | 0.08 ± 0.03 | 0.07 ± 0.03 | 0.78 ± 0.03 | 0.08 ± 0.01 |
| Heart | 0.96 ± 0.30 | 0.31 ± 0.04 | 0.30 ± 0.06 | 0.52 ± 0.05 | 0.16 ± 0.01 |
| Lung | 2.17 ± 0.70 | 0.72 ± 0.11 | 0.52 ± 0.04 | 1.20 ± 0.12 | 0.48 ± 0.12 |
| Spleen | 3.66 ± 0.87 | 2.82 ± 0.28 | 1.06 ± 0.18 | 1.88 ± 0.31 | 1.73 ± 0.38 |
| Kidneys | 4.44 ± 0.51 | 1.69 ± 0.09 | 1.65 ± 0.10 | 3.86 ± 0.28 | 1.36 ± 0.20 |
| Stomach | 1.78 ± 0.23 | 0.84 ± 0.19 | 0.72 ± 0.07 | 1.10 ± 0.26 | 0.54 ± 0.06 |
| pancreas | 0.94 ± 0.49 | 0.35 ± 0.03 | 0.39 ± 0.02 | 0.47 ± 0.03 | 0.19 ± 0.04 |
| Intestines | 1.88 ± 0.18 | 1.01 ± 0.04 | 1.07 ± 0.04 | 1.35 ± 0.12 | 0.78 ± 0.17 |
| Liver | 5.16 ± 0.99 | 4.90 ± 0.13 | 1.04 ± 0.10 | 6.19 ± 0.56 | 4.93 ± 0.30 |
| Muscle | 0.66 ± 0.26 | 0.31 ± 0.03 | 0.28 ± 0.04 | 0.38 ± 0.05 | 0.15 ± 0.05 |
| Bone | 1.17 ± 0.07 | 0.56 ± 0.03 | 0.52 ± 0.11 | 0.70 ± 0.15 | 0.32 ± 0.03 |
| Skin | 2.28 ± 0.39 | 1.23 ± 0.20 | 0.97 ± 0.06 | 1.48 ± 0.10 | 0.74 ± 0.11 |
| Brain | 0.12 ± 0.05 | 0.06 ± 0.01 | 0.06 ± 0.01 | 0.06 ± 0.01 | 0.03 ± 0.00 |
| U87MG Tumor | 4.88 ± 0.67 | 2.99 ± 0.16 | 3.00 ± 0.38 | 3.38 ± 0.37 | 2.35 ± 0.27 |
| Tumor-to-blood | 4.82 ± 1.60 | 40.1 ± 15.6 | 47.2 ± 18.0 | 4.34 ± 0.57 | 28.0 ± 4.26 |
| Tumor-to-liver | 0.99 ± 0.14 | 0.61 ± 0.04 | 2.90 ± 0.38 | 0.55 ± 0.09 | 0.48 ± 0.07 |
| Tumor-to-kidney | 1.16 ± 0.29 | 1.77 ± 0.13 | 1.82 ± 0.21 | 0.88 ± 0.07 | 1.74 ± 0.19 |

values shown represent the mean ± S.D. of data from three animals (n=3) per cohort

**Supplementary Table S3** Biodistribution of ^44^Sc-NODAGA-RGD and ^68^Ga-NODAGA-RGD in U87MG tumor-bearing female nude mice, expressed in percentage of total injected activity per gram tissue (% IA/g)

|  | ^44^Sc-NODAGA-RGD | | | ^68^Ga-NODAGA-RGD | |
| --- | --- | --- | --- | --- | --- |
|  | 30 min p.i. | 2 h p.i. | 5 h p.i. | 30 min p.i. | 2 h p.i. |
| Blood | 0.98 ± 0.21 | 0.12 ± 0.02 | 0.11 ± 0.07 | 1.12 ± 0.27 | 0.03 ± 0.01 |
| Heart | 0.89 ± 0.13 | 0.44 ± 0.04 | 0.35 ± 0.13 | 0.81 ± 0.12 | 0.23 ± 0.01 |
| Lung | 2.20 ± 0.68 | 1.10 ± 0.07 | 0.65 ± 0.16 | 1.69 ± 0.22 | 0.51 ± 0.07 |
| Spleen | 1.63 ± 0.21 | 1.58 ± 0.31 | 0.95 ± 0.19 | 1.64 ± 0.28 | 1.40 ± 0.30 |
| Kidneys | 3.89 ± 0.68 | 1.50 ± 0.30 | 1.16 ± 0.17 | 5.15 ± 0.72 | 2.11 ± 0.10 |
| Stomach | 1.69 ± 0.16 | 1.01 ± 0.32 | 0.80 ± 0.03 | 1.53 ± 0.08 | 0.58 ± 0.05 |
| pancreas | 0.73 ± 0.22 | 0.43 ± 0.04 | 0.37 ± 0.07 | 0.64 ± 0.11 | 0.23 ± 0.03 |
| Intestines | 1.76 ± 0.22 | 0.91 ± 0.16 | 0.75 ± 0.13 | 1.74 ± 0.12 | 0.94 ± 0.05 |
| Liver | 1.49 ± 0.06 | 1.34 ± 0.27 | 1.06 ± 0.16 | 2.68 ± 0.18 | 2.09 ± 0.08 |
| Muscle | 0.68 ± 0.03 | 0.42 ± 0.03 | 0.30 ± 0.06 | 0.55 ± 0.13 | 0.17 ± 0.03 |
| Bone | 1.53 ± 0.16 | 0.94 ± 0.10 | 0.76 ± 0.03 | 0.92 ± 0.07 | 0.42 ± 0.03 |
| Skin | 1.97 ± 0.17 | 1.30 ± 0.13 | 1.21 ± 0.34 | 2.05 ± 0.08 | 0.96 ± 0.01 |
| Brain | 0.10 ± 0.01 | 0.09 ± 0.01 | 0.06 ± 0.01 | 0.07 ± 0.01 | 0.03 ± 0.00 |
| U87MG Tumor | 4.50 ± 0.77 | 4.05 ± 0.89 | 3.01 ± 0.55 | 4.60 ± 0.59 | 3.13 ± 0.27 |
| Tumor-to-blood | 4.73 ± 1.24 | 34.1 ± 07.6 | 30.5 ± 9.93 | 4.17 ± 0.47 | 114 ± 35.0 |
| Tumor-to-liver | 3.02 ± 0.40 | 3.01 ± 0.13 | 2.82 ± 0.21 | 1.72 ± 0.16 | 1.50 ± 0.20 |
| Tumor-to-kidney | 1.18 ± 0.28 | 2.68 ± 0.14 | 2.58 ± 0.24 | 0.89 ± 0.03 | 1.49 ± 0.21 |

values shown represent the mean ± S.D. of data from three animals (n=3) per cohort

Biodistribution studies were performed with ^44^Sc and ^68^Ga labeled DOTA-NOC and NODAGA-NOC, respectively, in AR42J tumor-bearing mice (Supplementary Tables S4/S5).

**Supplementary Table S4** Biodistribution of ^44^Sc-DOTA-NOC and ^68^Ga-DOTA-NOC in AR42J tumor-bearing female nude mice, expressed in percentage of total injected activity per gram tissue (% IA/g)

|  | ^44^Sc-DOTA-NOC | | | ^68^Ga-DOTA-NOC | |
| --- | --- | --- | --- | --- | --- |
|  | 30 min p.i. | 2 h p.i. | 5 h p.i. | 30 min p.i. | 2 h p.i. |
| Blood | 2.91 ± 1.31 | 0.15 ± 0.02 | 0.11 ± 0.01 | 2.38 ± 0.20 | 0.26 ± 0.03 |
| Heart | 1.68 ± 0.76 | 0.22 ± 0.03 | 0.18 ± 0.07 | 1.06 ± 0.18 | 0.18 ± 0.02 |
| Lung | 3.43 ± 1.25 | 0.74 ± 0.05 | 0.47 ± 0.18 | 2.71 ± 0.43 | 1.02 ± 0.20 |
| Spleen | 0.93 ± 0.29 | 0.30 ± 0.04 | 0.27 ± 0.06 | 2.17 ± 0.22 | 2.13 ± 0.53 |
| Kidneys | 12.6 ± 3.36 | 8.72 ± 0.40 | 6.54 ± 0.30 | 10.3 ± 1.84 | 8.65 ± 0.75 |
| Adrenals | 2.82 ± 0.99 | 1.50 ± 0.24 | 1.36 ± 0.55 | 1.36 ± 0.27 | 0.77 ± 0.13 |
| Stomach | 1.38 ± 0.29 | 0.71 ± 0.05 | 0.52 ± 0.09 | 2.27 ± 0.21 | 1.68 ± 0.27 |
| Pancreas | 1.29 ± 0.28 | 0.71 ± 0.01 | 0.57 ± 0.07 | 2.48 ± 0.11 | 1.70 ± 0.22 |
| Intestines | 1.02 ± 0.37 | 0.30 ± 0.08 | 0.21 ± 0.03 | 0.99 ± 0.13 | 0.54 ± 0.08 |
| Liver | 1.49 ± 0.49 | 0.68 ± 0.07 | 0.36 ± 0.06 | 5.02 ± 0.26 | 5.52 ± 0.88 |
| Muscle | 0.68 ± 0.25 | 0.21 ± 0.01 | 0.15 ± 0.00 | 0.48 ± 0.07 | 0.07 ± 0.01 |
| Bone | 1.09 ± 0.22 | 0.25 ± 0.04 | 0.17 ± 0.04 | 0.86 ± 0.31 | 0.25 ± 0.08 |
| Brain | 0.10 ± 0.03 | 0.05 ± 0.00 | 0.05 ± 0.00 | 0.10 ± 0.01 | 0.02 ± 0.00 |
| AR42J Tumor | 9.49 ± 0.76 | 8.83 ± 0.57 | 5.56 ± 0.40 | 12.3 ± 3.14 | 12.2 ± 2.29 |
| Tumor-to-blood | 3.69 ± 1.52 | 58.2 ± 6.64 | 52.0 ± 4.38 | 5.22 ± 1.07 | 46.2 ± 2.28 |
| Tumor-to-liver | 6.87 ± 2.38 | 13.1 ± 2.16 | 15.7 ± 2.82 | 2.44 ± 0.36 | 2.22 ± 0.22 |
| Tumor-to-kidney | 0.79 ± 0.19 | 1.01 ± 0.06 | 0.85 ± 0.04 | 1.20 ± 0.21 | 1.41 ± 0.18 |

values shown represent the mean ± S.D. of data from three animals (n=3) per cohort

**Supplementary Table S5** Biodistribution of ^44^Sc-NODAGA-NOC and ^68^Ga-NODAGA-NOC in AR42J tumor-bearing female nude mice, expressed in percentage of total injected activity per gram tissue (% IA/g)

|  | ^44^Sc-NODAGA-NOC | | | ^68^Ga-NODAGA-NOC | |
| --- | --- | --- | --- | --- | --- |
|  | 30 min p.i. | 2 h p.i. | 5 h p.i. | 30 min p.i. | 2 h p.i. |
| Blood | 1.89 ± 0.08 | 0.22 ± 0.06 | 0.14 ± 0.01 | 2.07 ± 0.32 | 0.13 ± 0.01 |
| Heart | 2.27 ± 0.14 | 0.38 ± 0.10 | 0.30 ± 0.04 | 1.07 ± 0.12 | 0.11 ± 0.01 |
| Lung | 4.28 ± 0.03 | 1.19 ± 0.28 | 0.89 ± 0.14 | 2.69 ± 0.28 | 0.65 ± 0.04 |
| Spleen | 0.91 ± 0.20 | 0.38 ± 0.05 | 0.47 ± 0.05 | 0.91 ± 0.06 | 0.57 ± 0.16 |
| Kidneys | 12.4 ± 1.41 | 8.64 ± 1.62 | 13.0 ± 2.98 | 15.6 ± 3.13 | 11.5 ± 1.15 |
| Adrenals | 3.19 ± 0.22 | 2.37 ± 0.11 | 2.87 ± 0.95 | 1.51 ± 0.76 | 0.52 ± 0.14 |
| Stomach | 1.45 ± 0.28 | 1.26 ± 0.22 | 1.24 ± 0.09 | 1.52 ± 0.60 | 1.06 ± 0.20 |
| Pancreas | 1.60 ± 0.12 | 1.12 ± 0.07 | 1.35 ± 0.29 | 1.63 ± 0.11 | 1.11 ± 0.22 |
| Intestines | 0.74 ± 0.10 | 0.29 ± 0.07 | 0.38 ± 0.06 | 1.00 ± 0.15 | 0.44 ± 0.06 |
| Liver | 1.22 ± 0.11 | 0.88 ± 0.15 | 0.94 ± 0.12 | 2.09 ± 0.46 | 1.68 ± 0.19 |
| Muscle | 0.66 ± 0.08 | 0.30 ± 0.08 | 0.26 ± 0.07 | 0.43 ± 0.03 | 0.06 ± 0.01 |
| Bone | 1.38 ± 0.17 | 0.54 ± 0.12 | 0.46 ± 0.06 | 0.86 ± 0.25 | 0.16 ± 0.03 |
| Brain | 0.09 ± 0.01 | 0.06 ± 0.00 | 0.06 ± 0.01 | 0.08 ± 0.03 | 0.02 ± 0.01 |
| AR42J Tumor | 9.90 ± 0.66 | 10.2 ± 1.17 | 10.8 ± 0.37 | 8.85 ± 0.54 | 9.78 ± 0.54 |
| Tumor-to-blood | 5.23 ± 0.22 | 50.3 ± 16.8 | 78.6 ± 6.95 | 4.32 ± 0.58 | 76.0 ± 4.31 |
| Tumor-to-liver | 8.13 ± 0.72 | 11.3 ± 3.26 | 11.6 ± 1.51 | 4.36 ± 0.91 | 5.88 ± 0.68 |
| Tumor-to-kidney | 0.80 ± 0.10 | 1.22 ± 0.34 | 0.87 ± 0.24 | 0.58 ± 0.10 | 0.85 ± 0.08 |

values shown represent the mean ± S.D. of data from three animals (n=3) per cohort

**PET/CT Imaging**

In order to investigate how ^44^Sc and ^68^Ga would distribute within the body if they are injected as ions, PET/CT scans were performed with two mice without tumors, 5 h after injection of 10 MBq of ^44^ScCl_3_ (diluted in saline, pH ~4.5) and 10 MBq of ^68^GaCl_3_ (diluted in saline, pH ~4.5), respectively (Supplementary Fig. S1). Unspecific uptake of radioactivity was found in the liver and intestinal tract after injection of ^68^Ga^3+^ and ^44^Sc^3+^, respectively. Bone uptake was not observed after injection of ^44^Sc^3+^ (Supplementary Fig. S1 A/B). This was in contrast to the result after injection of ^68^Ga^3+^ which showed distinct accumulation in the bones, particularly in the spinal cord and joints (Supplementary Fig. S1 C/D).

**
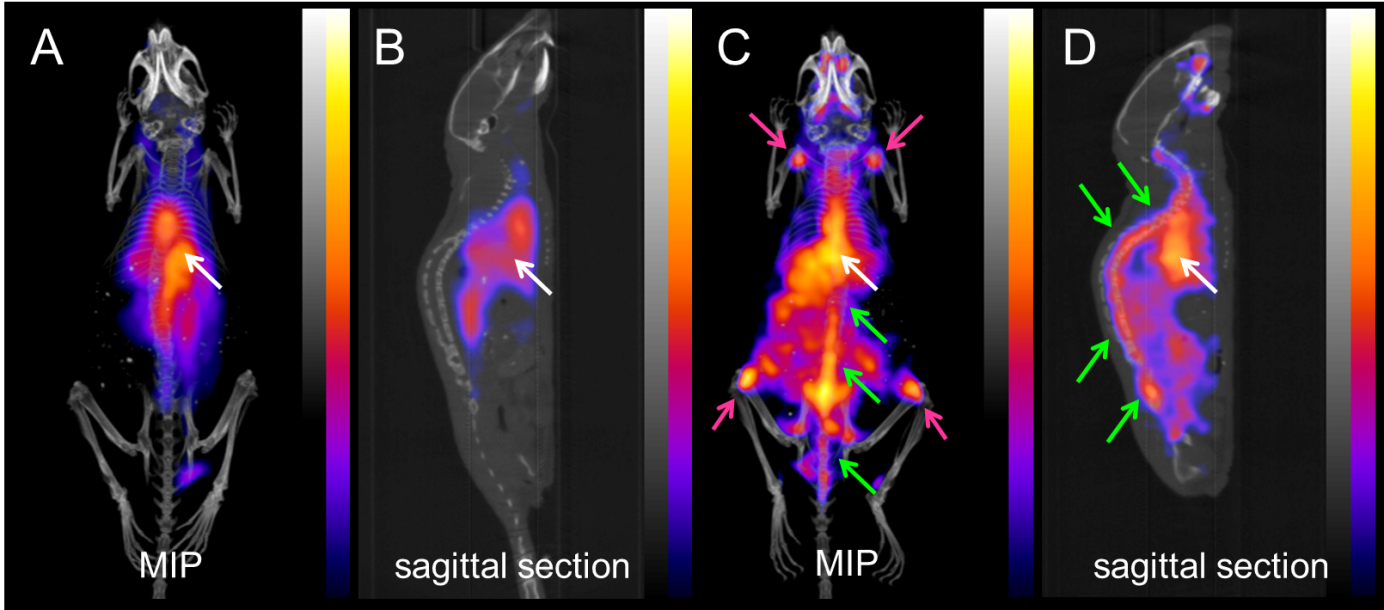
**

**Supplementary Fig. S1** PET/CT images of mice without tumors after injection of ^44^Sc^3+^ (**A/B**) and ^68^Ga^3+^ (**C/D**). Both radionuclides shows unspecific activity accumulation in the liver and intestines (white arrows). After injection of ^68^Ga^3+^ accumulation of radioactivity was also found in the spine (green arrows) and joints (pink arrows)

**References**

1. Müller C, Mindt TL, de Jong M, Schibli R. Evaluation of a novel radiofolate in tumour-bearing mice: promising prospects for folate-based radionuclide therapy. European journal of nuclear medicine and molecular imaging. 2009;36(6):938-46. doi:10.1007/s00259-008-1058-9.
